# Broadband acoustic subwavelength imaging by rapidly modulated stratified media

**DOI:** 10.1038/s41598-018-23411-5

**Published:** 2018-03-21

**Authors:** Xing-Feng Zhu, Qi Wei, Da-Jian Wu, Xiao-Jun Liu

**Affiliations:** 1 Key Laboratory of Modern Acoustics, Department of Physics and Collaborative Innovation Center of Advanced Microstructures, Nanjing University, Nanjing, 210093 China; 2 Jiangsu Key Lab on Opto-Electronic Technology, School of Physics and Technology, Nanjing Normal University, Nanjing, 210023 China

## Abstract

An acoustic anisotropic lens (AAL) based on large mass-density modulation depth (LMMD) medium is proposed for subwavelength imaging. The underlying mechanism for converting evanescent components into propagating waves is attributed to the strong suppression of the transverse velocity field component in LMMD medium. In addition, the proposed lens can operate in a broadband manner, which is more flexible in practical applications. Both transfer matrix method and finite element method are used to corroborate the subwavelength imaging capabilities of the proposed lens. The numerical simulations demonstrate that the proposed lens can clearly distinguish two Gaussian sources with equal width of λ_0_/25 and separation of λ_0_/5 in a broad frequency bandwidth. Medium losses decrease the transmission but cannot compromise the resolution of the lens.

## Introduction

Subwavelength imaging of acoustic waves has been a topic of growing interest because of their potential applications in medicine and detection^1,2^. To get a subwavelength image, the key point is manipulating evanescent waves, which carry the subwavelength details of the objects. In recent years, much attention has been paid to design the metamaterial lens for enhancing or maintaining the evanescent fields^3–5^. One example is acoustic lenses made of doubly negative mass density and bulk modulus^4,6^ or single negative mass density metamaterials^7–10^, in which the evanescent waves are resonantly enhanced due to the couplings with the surface state. However, only parts of evanescent waves in the *k* space can couple with the surface modes and hence the field enhancements are non-uniform with respect to the spatial frequencies. Further works focused on anisotropic acoustic metamaterials, where evanescent wave amplitudes can be uniformly enhanced due to their hyperbolic or nearly flat equifrequency contours (EFCs)^11–18^. A deep subwavelength resolution down to $\lambda /50$ was achieved by a lens made of a three-dimensional holey-structured anisotropic metamaterial^15^. However, the evanescent waves must couple with the Fabry–Pérot (FP) resonant condition to achieve efficient transmission. Therefore, the working frequency ranges in these FP resonant lenses are relatively narrow, which restricts the practically achievable imaging.

Recently, a rapidly modulated stratified media has been proposed to achieve subwavelength imaging in optics^19,20^. The large modulation of permittivity strongly suppresses the longitudinal component of the electric field (${E}_{z}\to 0$), inducing the infinite effective permittivity (${\varepsilon }_{z}\to \infty$). Thus, the EFC in such strongly anisotropic medium is nearly flat and the evanescent waves can be converted into propagating waves and then transferred across the metamaterial lens, forming an image. Inspired by the optical subwavelength imaging phenomena, subwavelength imaging of acoustic waves may occur in the modulated stratified medium with a large mass-density modulation depth (LMMD) medium. The LMMD medium can be regarded as an acoustic metamaterial with the uniform effective mass density and has nearly flat EFC^21^. In this paper, we propose an acoustic anisotropic lens (AAL) based on the (LMMD) medium to realize subwavelength imaging. Transfer matrix method (TMM) is employed to investigate the subwavelength images properties of the AAL. It is demonstrated that the proposed AAL can clearly distinguish two deep subwavelength (${\lambda }_{0}/25$) sources with a separation of ${\lambda }_{0}/5$ both in lossless and lossy cases. Importantly, the AAL can operate over a broad frequency bandwidth. Finite element method (FEM) is conducted to further verify the results.

## Results

### AAL based on LMMD medium

**Figure 1:**
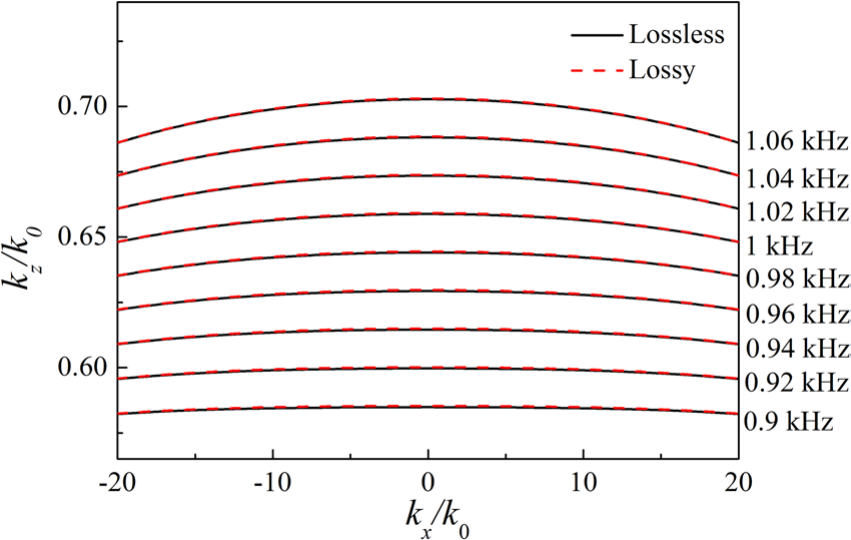
EFCs of the LMMD medium at different frequencies.

The LMMD medium is composed of stratified media with a period of $\Lambda$ ($\Lambda =\eta {\lambda }_{0}$, ${\lambda }_{0}$ is the incident wavelength and *η* is a small parameter). The unit cell comprises *N* homogeneous layers with thicknesses of $\Lambda /N$ and the mass density of *j*th $(j=1,\ldots ,N)$ layer is defined as

1 $${\rho }_{j}={\rho }_{0}{\rho }_{r}+{\rho }_{0}(1/\eta +i\delta \rho )\cos [\frac{2\pi }{N}(j-1)],$$

where ${\rho }_{0}{\rho }_{r}$ is the mean value of the mass density, and ${\rho }_{0}\delta \rho$ (not large) is responsible for the modulation of medium absorption. As the modulation of the mass density (*η* is small) becomes large in comparison with the mean value, the acoustic wave evolution will be affected not only by the value of the mass density, but also by additional important contributions arising from the rapidly-varying periodic density oscillations, which will strongly suppress the transverse component of the velocity field. The LMMD medium can be regarded as an acoustic metamaterial with the uniform effective mass density. Therefore, the propagating of acoustic waves in the LMMD medium can be simulated by that in an effective homogeneous medium^21^. Based on the LMMD medium, we propose an acoustic anisotropic lens (AAL). The AAL is constructed by a LMMD medium slab with a subwavelength thickness $L=45\Lambda$. The structural parameters are determined at frequency ${f}_{0}=1$ kHz. Here, *N* = 10 and *η* = 1/60. The surface of the lens is parallel to *x*-axis and perpendicular to *z*-axis. The equifrequency contours for acoustic metamaterials with anisotropic mass can be described by

2 $$\frac{{k}_{x}^{2}}{{\rho }_{x}}+\frac{{k}_{z}^{2}}{{\rho }_{z}}=\frac{{\omega }^{2}}{B},$$

where ${k}_{x}$ and ${k}_{z}$ are the wave vectors parallel and perpendicular to the slab surface, respectively, and *B* is the bulk modulus. In the LMMD medium, the transverse component of the velocity field is strongly suppressed, *i*.*e*. ${v}_{x}\to 0$ and then ${\rho }_{x}\to \infty$. The infinite ${\rho }_{x}$ in the LMMD medium is different from the ones in previous anisotropic metamaterials, which arises from the extremely large impedance mismatch between the acoustically hard material and the surrounding fluid^15–18^. Then, the evanescent waves could propagate with the same wave vector ${k}_{z}=\omega \sqrt{{\rho }_{z}/B}$ according to Eq. (2). Figure 1 shows the EFCs of the LMMD medium at different frequencies. The EFCs are obtained by imposing the Bloch condition on the field amplitudes evaluated through the TMM. The solid and dashed lines represent the EFCs for the lossless LMMD medium with ${\rho }_{m}=0.05{\rho }_{0}$, $\delta \rho =0$ and lossy LMMD medium with ${\rho }_{m}=(0.05+0.02i){\rho }_{0}$, $\delta \rho =0.01{\rho }_{0}$, respectively. It is clear that the EFCs of the two cases are almost the same and very flat for a large range of wavevectors in frequency range from 0.9 to 1.06 kHz. The long and flat EFCs indicate that the evanescent waves can be converted into propagating waves and then transferred across the LMMD medium. Therefore, the subwavelength details of the source can be preserved inside the AAL.

### Numerical demonstrations

**Figure 2:**
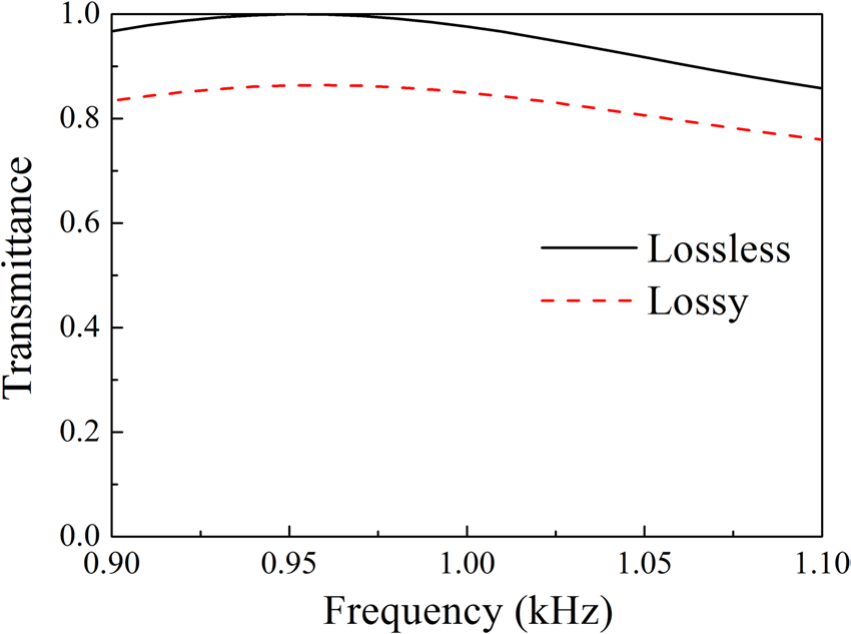
Transmittance spectra for acoustic waves through the AAL.

We first investigate the transmission efficiency of the AAL with different frequencies. Figure 2 shows the transmission spectra of the AAL calculated by using the TMM. Here, acoustic waves are normally incident on the AAL. The frequency range is chosen from 0.9 kHz to 1.1 kHz, which fluctuates $\pm 0.1$ kHz at the working frequency ${f}_{0}$. The solid line represents the transmission spectra of the AAL for the lossless case with ${\rho }_{r}=0.05$ and $\delta \rho =0$. It is obvious that the unit transmitted amplitude can be obtained near the working frequency and the transmitted amplitudes are larger than 0.85 in the frequency range from 0.9 to 1.1 kHz. The dash line represents the transmission spectra of the AAL for the lossy case with ${\rho }_{r}=0.05+0.02i$ and $\delta \rho =0.01$. The transmitted amplitudes are larger than 0.75 over the frequency range. Compared to the lossless case, the transmittance in the lossy case decreases due to the medium absorption. Therefore, the AAL has a very high efficiency over the frequency range both in the lossless and lossy cases.

Then, we check the subwavelength imaging features of the AAL. The modulation transfer function (MTF) can be obtained according to the ratio of the output spatial spectra to the incident fields,

3 $$\hat{p}({k}_{x},z=L)={\rm{MTF}}({k}_{x})\cdot {\hat{p}}^{inc}({k}_{x},z=0).$$

The point spread function (PSF) is the inverse Fourier transform of the MTF. The transmitted acoustic wave field profile $p(x,L)$ can be expressed as the convolution of PSF(*x*) with the incident acoustic wave field $p(x,0)\,$^22^, i.e.,

4 $$p(x,z=L)={\rm{PSF}}(x)\ast p(x,z=0).$$

**Figure 3:**
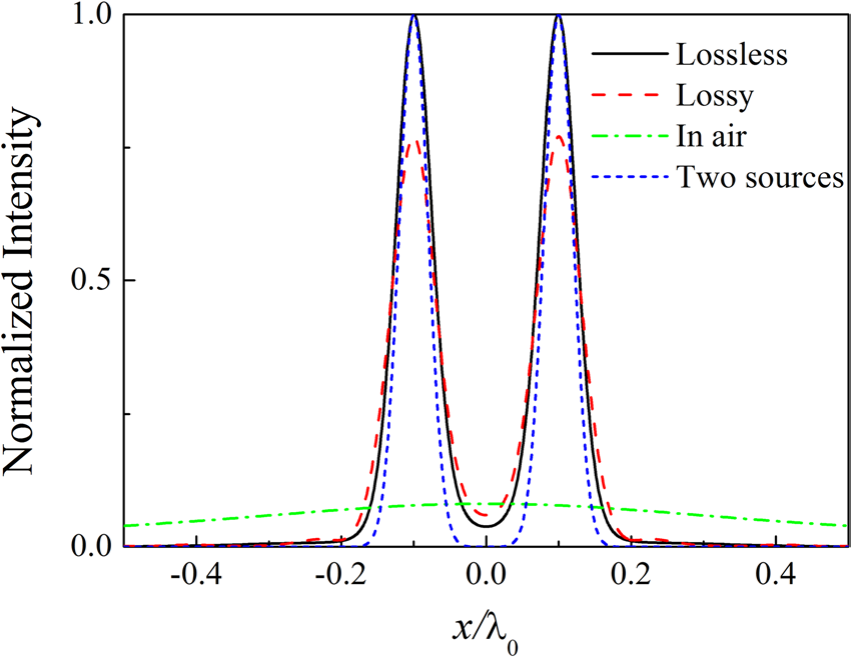
Acoustic intensity profile of images along *x* direction on the output surface of the AAL in the lossless and lossy cases based on TMM. The dotted line represents the two deep subwavelength Gaussian sources. The dash-dotted line represents the intensity profile at the same position for the case without the lens.

In Fig. 3, we show the comparison between the output images and input sources. The sources are composed of two Gaussians with an equal width of ${\rm{\sigma }}={\lambda }_{0}/25$ and the separation is $d={\lambda }_{0}/5$ (center-to-center), i.e.,

5 $$p={p}_{0}({e}^{-{(x+2.5{\rm{\sigma }}/2)}^{2}/{{\rm{\sigma }}}^{2}}+{e}^{-{(x-2.5{\rm{\sigma }}/2)}^{2}/{{\rm{\sigma }}}^{2}}).$$

Here, ${p}_{0}=1$ Pa. The solid and dashed lines represent the output images in the lossless and lossy cases, respectively. The dotted line represents the two deep subwavelength Gaussian sources. The output images with the lossless AAL are almost the copy of the input ones. In the presence of losses, the output images appear at the price of reduced amplitude and a slower decay far from the sources, but the resolution is only little affected. To appreciate such an imaging result, we also show the effect of air propagation on the above incoming field in Fig. 3 (dash-dotted line), and the complete deterioration of the considered subwavelength image is evident. Thus, sub-wavelength imaging is possible for the lossless or lossy AAL based on LMMD medium.

**Figure 4:**
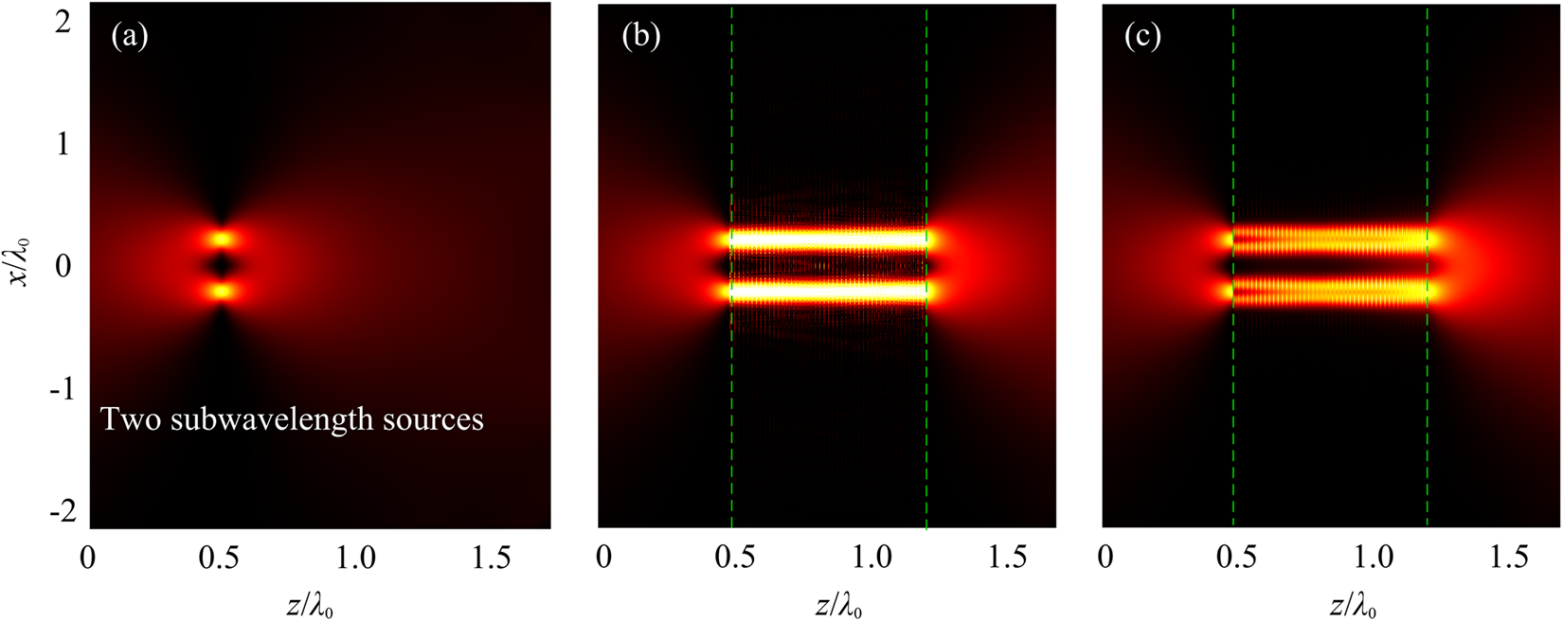
(**a**) Acoustic intensity distribution of two subwavelength sources propagating in air. Acoustic intensity distribution of two subwavelength sources for (**b**) lossless lens and (**c**) lossy lens. The lens has a thickness $L=0.75{\lambda }_{0}$. The thicknesses of the air layers in the two sides of the lens are both set as 0.5${\lambda }_{0}$. The dashed lines indicate the interfaces between air and lens.

**Figure 5:**
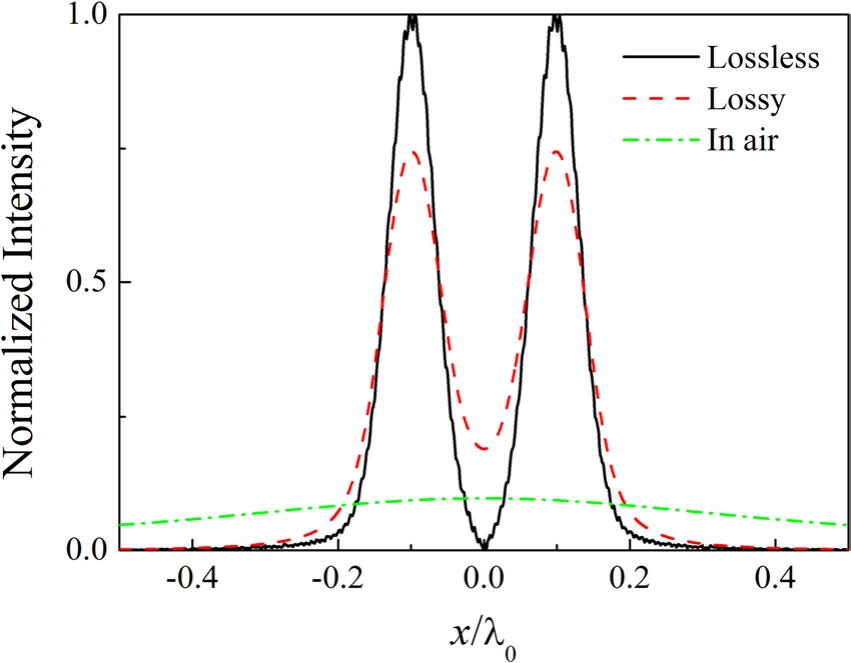
Acoustic intensity profile of images along *x* direction on the output surface of the AAL in the lossless and lossy cases based on FEM. The dash-dotted line represents the intensity profile at the same position for the case without the lens.

We further demonstrate subwavelength imaging of the AAL by using the full-wave simulations based on the finite element method (FEM). The acoustic intensity distributions of the two subwavelength sources are shown in Fig. 4. The material parameters of the lens are the same as those of Fig. 3. The thicknesses of the air layers at the two sides of the lens are both set as 0.5${\lambda }_{0}$. Periodic boundary conditions are imposed in the *x* direction, and the radiation boundary conditions are set for the remaining boundaries. The two sources are placed near the input surface of the lens. Figure 4(a) shows the acoustic intensity distribution of the sources without AAL. The subwavelength information is lost in the far-field due to the diffraction limit. Figure 4(b,c) show the acoustic intensity distributions for the lossless and lossy AALs, respectively. It is apparent that in contrast to the case without the lens, the subwavelength details are preserved on the output surface when the lens is installed. The source is directly transferred within a pipeline in the *z* direction resulting from the non-diffraction feature of the LMMD medium. Hence, the two deep subwavelength sources are clearly resolved after the source signals pass through the lens. The AAL based on LMMD medium presents an infinitely anisotropic structure that allows wave propagation in the direction perpendicular to the interfaces. To show the imaging effects more clearly, Fig. 5 gives the normalized acoustic intensity profile of images along *x* direction on the output surface of the AAL. The solid and dashed lines represent the output images for the lossless and lossy lenses, respectively. The dash-dotted line represents the intensity profile at the same position but in the absence of the lens, i.e., propagation in air after the distance of *L*. It can be found that the images at the output of the lens are clearly resolved in sharp contrast to the results obtained in the absence of the lens. The complete deterioration of the considered subwavelength image is evident after propagating the distance of *L* in air. Medium losses decrease the amplitude of the images but cannot compromise the resolution of the lens. In Fig. 5, the results obtained by using FEM are in well agreement with those found in Fig. 3. Therefore, the AAL can faithfully project an image with deep-subwavelength details (${\lambda }_{0}/25$) and clearly distinguish two sources with separation of ${\lambda }_{0}/5$ both in the lossless and lossy cases.

**Figure 6:**
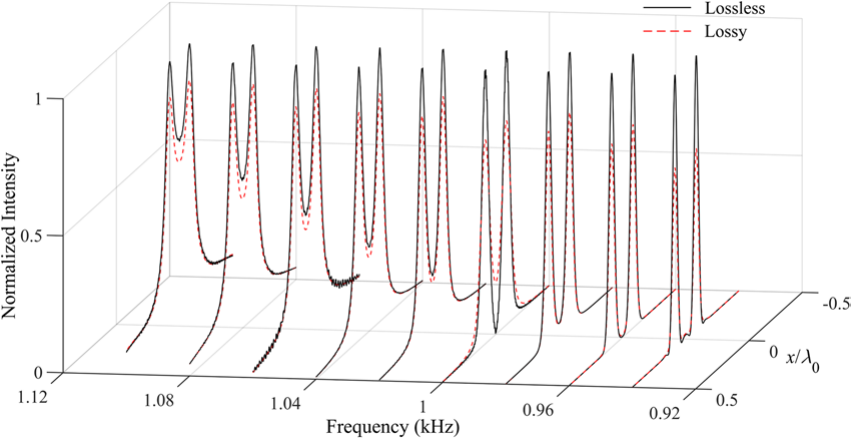
Broadband performance of the AAL. Normalized intensity along *x* direction on the output surface of the AAL as a function of frequency.

To examine the frequency response property of the AAL, we use the same setup as used in Fig. 4(b,c) to perform the simulation at different frequencies. Figure 6 shows the broadband response of the lens for frequencies from 0.94 to 1.1 kHz. The solid and dashed lines represent the normalized intensity profile at each frequency along *x* direction on the output surface for the lossless and lossy AALs, respectively. The two sources can be clearly resolved throughout the frequency range although the lens is designed for a specific frequency. Deviating from the specific frequency ${f}_{0}$, the subwavelength imaging efficiency is evident even in the presence of a slight image deterioration resulting from the side lobes. Medium losses decrease the transmitted amplitude but little affect the resolution of the lens throughout the frequency range. Therefore, the broadband subwavelength imaging can be realized with the AAL and the imaging is robust against the material losses.

## Discussion

The AAL based on LMMD medium has been proposed to realize acoustic subwavelength imaging. Different from the anisotropic or high-contrast media^23–26^ based on resonant mechanism, the proposed lens can operate in a broadband without the limitation on working frequency, which is more flexible in practical applications. The infinite ${\rho }_{x}$ in AAL lens is attributed to the strong suppression of the transverse velocity field component in LMMD medium. Thus, the EFCs in AAL lens are very flat and the subwavelength source can be directly transferred within a pipeline in the lens. The AAL can faithfully project an image with deep-subwavelength details (${\lambda }_{0}/25$) and clearly distinguish two sources with separation of ${\lambda }_{0}/5$. Moreover, the subwavelength imaging is unhampered by the material losses throughout the frequency range. This feature is very important in the subwavelength imaging since material losses are always present and will generally weaken the effect of lens. Applications based on such metamaterial lens can be anticipated in the areas of ultrasonic medical imaging and non-destructive evaluation.

## Methods

Our numerical analysis combines two elements: the TMM, which is used to calculate the transmission of a single plane wave through the lens, and the theory of linear shift-invariant systems (LSI)^27,28^, which gives the description of the multilayer with the MTF or PSF. LSI are well known in different fields of optics. Here we apply this model to analyze the acoustical stratified media in a situation when they act as imaging elements for coherent monochromatic acoustic wave. We study separately the transmission of plane waves with different real values of the wavevector component *k*_*x*_, corresponding to both propagating and evanescent waves. Our present analysis is limited to transmission through multilayers surrounded by air and does not include a source and detector. Apart from TMM, numerical simulations are performed by the commercial software, Comsol Multiphysics, which is based on the FEM. The background medium is air with a mass density ${\rho }_{0}=1.25$ kg/m^3^ and a sound speed ${c}_{0}=343\,\,$m/s. Periodic boundary conditions are imposed in the *x* direction, and the radiation boundary conditions are set for the remaining boundaries. The largest mesh element size is lower than one tenth of the incident wavelength, and the further refined meshes are applied in the domain of the unit cells of the microstructure.

## References

[1] Hildebrand JA (1981). C. F. Acoustic microscopy of living cells. Proc. Natl. Acad. Sci. USA.

[2] Sackmann M (1988). Shock-wave lithotripsy of gallbladder stones. The first 175 patients. N. Engl. J. Med..

[3] Zhang X, Liu ZW (2008). Superlenses to overcome the diffraction limit. Nature Mater..

[4] Zhang S, Yin L, Fang N (2009). Focusing ultrasound with an acoustic metamaterial network. Phys. Rev. Lett..

[5] Christensen J, Garcia de Abajo FJ (2012). Anisotropic Metamaterials for Full Control of Acoustic Waves. Phys. Rev. Lett..

[6] Lee SH (2010). Composite Acoustic Medium with Simultaneously Negative Density and Modulus. Phys. Rev. Lett..

[7] Ambati M (2007). Surface resonant states and superlensing in acoustic metamaterials. Phys. Rev. B.

[8] Deng K (2009). Theoretical study of subwavelength imaging by acoustic metamaterial slabs. J. Appl. Phys..

[9] Park JJ (2015). Acoustic superlens using membrane-based metamaterials. Appl. Phys. Lett..

[10] Kaina N (2015). Negative refractive index and acoustic superlens from multiple scattering in single negative metamaterials. Nature.

[11] Torrent D, Sanchez-Dehesa J (2008). Anisotropic mass density by two-dimensional acoustic metamaterials. New J. Phys..

[12] Li J (2009). Experimental demonstration of an acoustic magnifying hyperlens. Nat. Mater..

[13] Jia H (2010). Subwavelength imaging by a simple planar acoustic superlens. Appl. Phys. Lett..

[14] Zhou X, Hu G (2011). Superlensing effect of an anisotropic metamaterial slab with near-zero dynamic mass. Appl. Phys. Lett..

[15] Zhu J (2011). A holey-structured metamaterial for acoustic deep-subwavelength imaging. Nat. Phys..

[16] Park CM (2011). Amplification of acoustic evanescent waves using metamaterial slabs. Phys. Rev. Lett..

[17] Cheng Y (2013). Acoustic subwavelength imaging of subsurface objects with acoustic resonant metalens. Appl. Phys. Lett..

[18] Amireddy KK, Balasubramaniam K, Rajagopal P (2016). Holey-structured metamaterial lens for subwavelength resolution in ultrasonic characterization of metallic components. Appl. Phys. Lett..

[19] Rizza C, Ciattoni A (2013). Effective medium theory for Kapitza stratified media: diffractionless propagation. Phys. Rev. Lett..

[20] Rizza C, Ciattoni A (2013). Kapitza homogenization of deep gratings for designing dielectric metamaterials. Opt. Lett..

[21] Zhu XF (2017). Non-diffraction propagation of acoustic waves in a rapidly modulated stratified medium. Sci. Rep..

[22] Kotyński R, Stefamiuk T (2009). Comparison of imaging with sub-wavelength resolution in the canalization and resonant tunnelling regimes. J. Opt. A: Pure Appl. Opt..

[23] Ammari H, Zhang H (2017). Effective medium theory for acoustic waves in bubbly fluids near Minnaert resonant frequency. SIAM J. Math. Anal..

[24] Ammari H, Zhang H (2015). Super-resolution in high-contrast media. Proc. R. Soc. A.

[25] Ammari H (2017). Sub-wavelength focusing of acoustic waves in bubbly media. Proc. R. Soc. A.

[26] Ammari H, Zhang H (2015). A mathematical theory of super-resolution by using a system of sub-wavelength Helmholtz resonators. Commun. in Math. Phys..

[27] Saleh, B. & Teich, M. Fundamentals of Photonics (New York: Wiley) (1991).

[28] Goodman, J. W. Introduction to Fourier Optics (New York: McGraw-Hill) (1996).

